# Nonconventional Use of Flash-Lamp Pulsed-Dye Laser in Dermatology

**DOI:** 10.1155/2016/7981640

**Published:** 2016-08-18

**Authors:** Steven Nisticò, Piero Campolmi, Silvia Moretti, Ester Del Duca, Nicola Bruscino, Rossana Conti, Andrea Bassi, Giovanni Cannarozzo

**Affiliations:** ^1^Department of Dermatology, University of Rome “Tor Vergata”, Viale Oxford 81, 00133 Rome, Italy; ^2^Department of Dermatology, University of Florence, Florence, Italy; ^3^Laser in Dermatology Unit, University of Rome “Tor Vergata”, Rome, Italy

## Abstract

Flash-lamp pulsed-dye laser (FPDL) is a nonablative technology, typically used in vascular malformation therapy due to its specificity for hemoglobin. FPDL treatments were performed in a large group of patients with persistent and/or recalcitrant different dermatological lesions with cutaneous microvessel involvement. In particular, 149 patients (73 males and 76 females) were treated. They were affected by the following dermatological disorders: angiokeratoma circumscriptum, genital and extragenital viral warts, striae rubrae, basal cell carcinoma, Kaposi's sarcoma, angiolymphoid hyperplasia, and Jessner-Kanof disease. They all underwent various laser sessions. 89 patients (59.7%) achieved excellent clearance, 32 patients (21.4%) achieved good-moderate clearance, 19 patients (12.7%) obtained slight clearance, and 9 subjects (6.1%) had low or no removal of their lesion. In all cases, FPDL was found to be a safe and effective treatment for the abovementioned dermatological lesions in which skin microvessels play a role in pathogenesis or development. Further and single-indication studies, however, are required to assess a standardized and reproducible method for applying this technology to “off-label” indications.

## 1. Introduction 

Flash-lamp pulsed-dye laser (FPDL) is a nonablative laser technology that has gained an excellent reputation in the treatment of vascular lesions. It uses a rhodamine dye that is dissolved in a solvent and pumped by a flash-lamp producing an emission of a 595 nm wavelength, approximately near to the hemoglobin's and oxyhemoglobin's absorption peaks. It is therefore considered the most specific laser currently available for the treatment of superficial vascular lesions. Current indications of this technology have further been extended to include nonvascular lesions but with vascular structural involvement, which makes them amenable to being treated with such laser.

FPDL does not always represent the first-line treatment for all these lesions: in fact, many lesions can be successfully treated using different treatment methods, and others (viral warts and molluscum contagiosum) may sometimes disappear without external intervention; therefore, the specification of when FPDL could be beneficial is crucial. Dermatological lesions that represent the target may be considered typical vascular lesions, vascular dependent lesions, or nonvascular lesions, according to a recent classification [1].

Vascular lesions include not only port-wine stains, superficial hemangiomas, and telangiectasias in which dye laser is considered the gold standard therapy but also angiokeratomas and lesions typical of the Bourneville-Pringle syndrome that may be treated surgically or through cryotherapy.

Vascular dependent lesions can be divided into the following: viral infections such as verruca vulgaris and genital viral warts, inflammatory skin diseases such as example localized psoriasis and lupus erythematosus, connective tissue diseases such as hypertrophic scars, keloids, and striae rubrae, neoplastic dermatosis such as basal cell carcinoma, Kaposi's sarcoma and, angiolymphoid hyperplasia.

Nonvascular lesions, on the other hand, include viral infections like molluscum contagiosum as well as other cutaneous conditions, for example, xanthelasma palpebrarum [1–13].

We selected a number of vascular dependent lesions and nonvascular lesions in an open study in order to verify the outcome of FPDL treatments.

## 2. Materials and Methods

**Figure 1:**
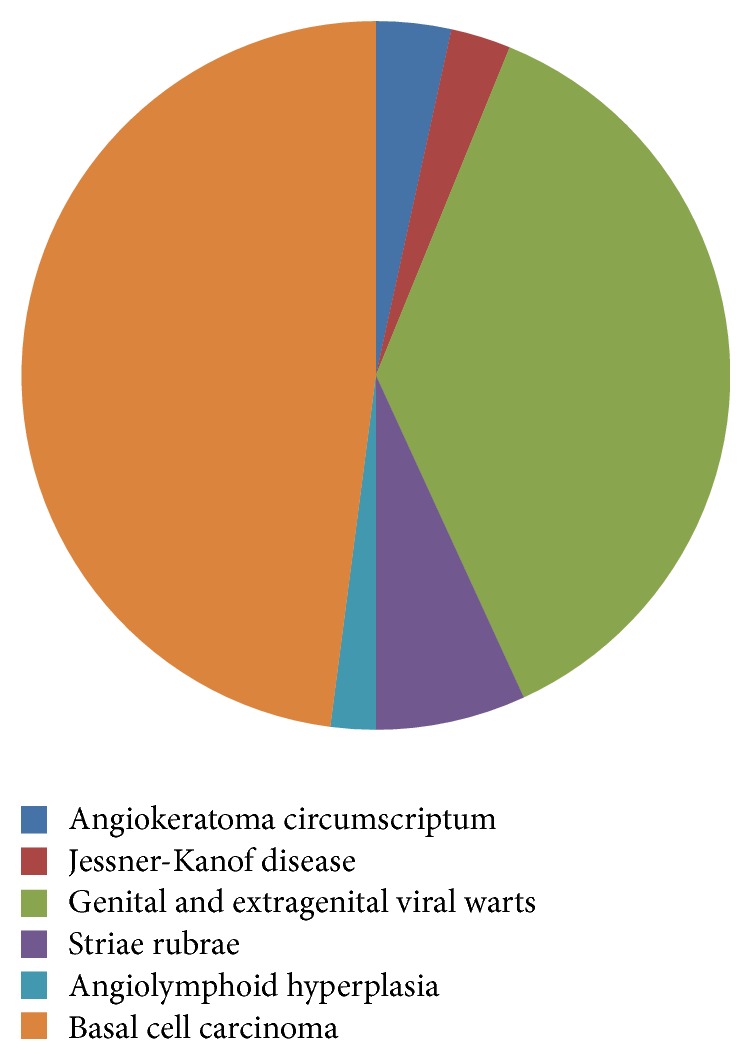
Type of lesions.

A total of 149 patients affected by angiokeratoma circumscriptum (5), Jessner-Kanof disease (4), genital and extragenital viral warts (54), striae rubrae (10), angiolymphoid hyperplasia (3), basal cell carcinoma (70), and Kaposi's sarcoma (3) were selected for treatment.  Figure 1.

Patients underwent different treatment sessions with the FPDL (Synchro Vas-Q, Deka MELA, Florence, Italy) as indicated by Table 1.

All patients underwent treatment after obtaining a detailed personal history (skin type, clinical manifestations, health conditions, previous medications, and life-style) and informed consent.

Five cases of angiokeratoma circumscriptum with presence of asymptomatic warty, keratotic nodules localized on the external genitals of adult men were treated using a 10600 nm CO_2_ laser (Smartxide^2^ Dot/RF, DEKA MELA, Florence), at 0.2–0.5 W, 10 Hz in order to obtain vaporization prior to three FPDL sessions.

**Figure 2:**
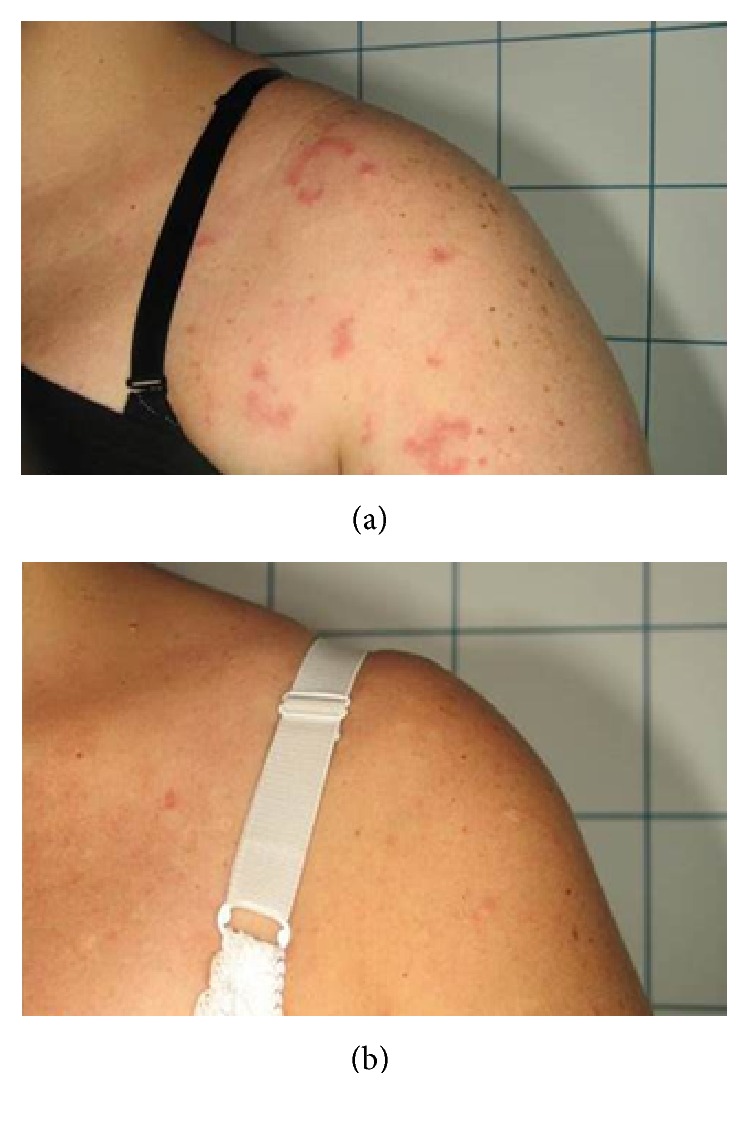
(a) A patient affected by Jessner-Kanof disease at baseline. (b) The disappearance of the lesions 12 months after the last PDL session.

Four patients with histological diagnosis of Jessner-Kanof disease, a benign yet chronic lymphocytic skin disorder, were treated in four FPDL laser sessions (Figure 2).

**Figure 3 fig3:**
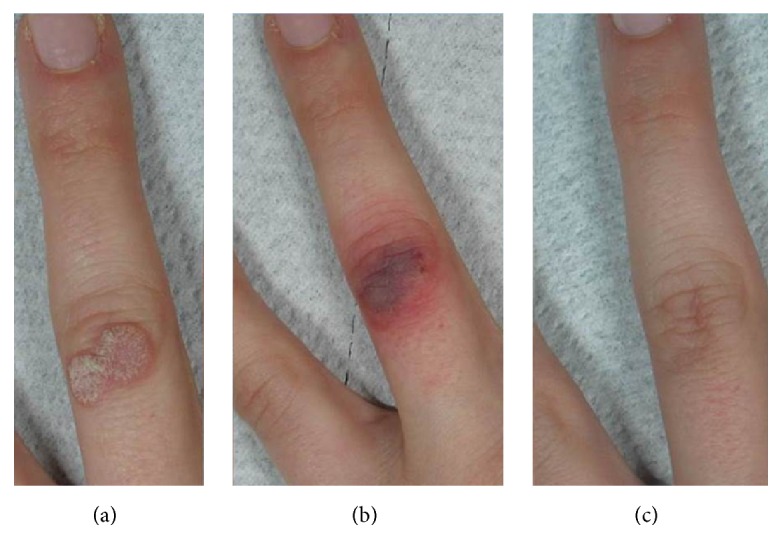
(a) A viral wart of the finger at baseline. (b) The typical immediate purpura after the PDL therapeutical session. (c) The excellent result achieved after 5 PDL treatments.

Fifty-four patients affected from viral warts (30 males and 24 females), between the ages of 6 and 75 years (mean 34.39 years old), were recruited with a total of 85 warts. The majority were children (range 6–18 years old) with localization on the hands (20 patients); in particular, lesions were located on the hands (palms, dorsum, and the fingertips), the periungual and subungual region, on the feet (12 patients) especially on the soles, in one case, on the fifth finger, and on the face (2 patients). Twelve of the older patients presented lesions on the periungual and subungual regions and eight of them had genital warts. All warts underwent at least one prior established treatment without success. All patients in the study were treated with the flash-lamp pumped-dye laser (three to seven treatment sessions every four weeks), preceded by curettage of the typical hyperkeratosis through salicylic acid 30% ointment, especially for the mosaic plantar warts (Figure 3).

Ten patients with striae rubrae were also treated with the FPDL. Four to six sessions of FPDL were employed in the treatment of striae which had the following localizations: abdomen/groin (3 patients), axilla/anterior shoulder (4 patients), and buttock/upper thigh (3 patients).

Three young females affected by angiolymphoid hyperplasia whose diagnosis was based on clinical and histological findings were selected in the present study. Patients received a previous radiotherapy with no results. In all these cases, patients were treated in three sessions using FPDL in addition to ablative CO_2_ laser (0.5–0.7 W) in order to obtain complete vaporization of the lesions.

**Figure 4 fig4:**
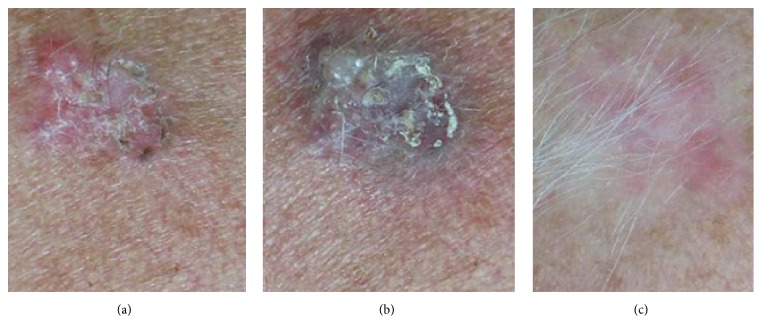
(a) A superficial basal cell carcinoma from the back of a woman at baseline. (b) The typical purpura due to PDL. (c) The disappearance of the lesion 12 months after the last PDL session.

Seventy patients (38 males and 32 females, aged 47 to 78) with superficial and nodular nonpigmented basal cell carcinoma (BCC), diagnosed after dermoscopic evaluation, were recruited. Most parts of the lesions were characterized by limited size (diameter < 1 cm) (Figure 4). The basal cell carcinomas were localized mainly on the face and neck area and in anatomically difficult areas as the nasal wings or the periocular zone. One patient had an allergy to anesthetics and five were cardiopathic, which limited the usage of surgical excision. All patients were subject to FPDL in five sessions with a 595 nm wavelength that enabled deeper penetration to the target (oxyhemoglobin or nuclear chromatin).

Three patients with classic Kaposi's sarcoma were treated with four FPDL sessions. The reason for treatments was due to the inability to undergo surgery or poor response to pharmacological treatments.

A summary of the methodology used for the different indications is provided in Table 1.

Laser fluence settings for all diseases were 6–8 J/cm^2^, a spot size of 12 mm, and a pulse duration of 0.5–1.5 ms.

Most of the lesions were treated without anesthesia. Its use has been limited as the procedure itself was not so painful and also because local anesthesia could cause oedema and hinder the “visual feedback processing” during treatment. An effective cooling device was always used during each laser session, improving comfort. Patients were instructed to avoid sun and cosmetics during the immediate postprocedural periods and to apply cool gauzes, emollient creams, and sunscreens until complete recovery. Daily application of cool wraps was useful to prevent the appearance of vesicles and blisters. An antibiotic ointment, gentamicin 0.1%, was also requested to be applied to the target areas for 7 days after each laser session, avoiding potential cutaneous superinfections.

## 3. Results

Results obtained were judged immediately and 4 weeks after the last session; treatment outcome was assessed by ranking the results into four categories, a quartile scale, of lesion clearance in comparison to baseline: 1 indicates no or low results (0–25% of the lesion area cleared), 2 indicates slight clearance (25–50% of the lesion area cleared), 3 indicates moderate-good clearance (50–75%), and 4 indicates excellent clearance (75%–100%).

Patients were asked for a subjective evaluation of the perceived overall results by means of the following score: unsatisfied, not very satisfied, satisfied, and very satisfied.

**Figure 5:**
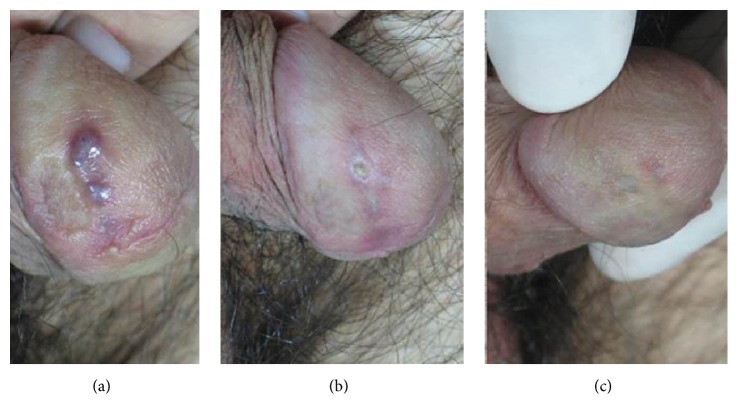
(a) Kaposi's sarcoma of the penis, baseline. (b) A temporary little ulcer after 3 PDL sessions. (c) The final result 12 months after the last PDL session, with no side effects and a great satisfaction of the patient.

All patients observed global improvements (Figures 2 [Fig fig3] [Fig fig4]–5 (a), (b), and (c)). All the lesions were completely removed except in the case of 7 patients with striae rubrae where lesions did not disappear at 12-month follow-up and two cases of BCC which showed a recurrence that was completely excised through surgery four weeks later.

102 patients (68.4%) achieved excellent clearance, 23 patients (15.4%) achieved good-moderate clearance, 15 patients (10%) obtained slight clearance, and 9 subjects (6%) had low or no removal of their lesion (Table 2).

Patients were asked for a subjective evaluation of the results: 110 patients (73.8%) were very satisfied, 23 (15.4%) were satisfied, and 14 (9.3%) were not very satisfied with the results, whereas only 2 patients (1.3%) were unsatisfied (Table 3); the low satisfaction rate was due probably to long-term purpura produced by the use of higher parameters which caused discomfort; dissatisfaction with the results was due to higher expectations in case of 2 patients with striae.

**Figure 6:**
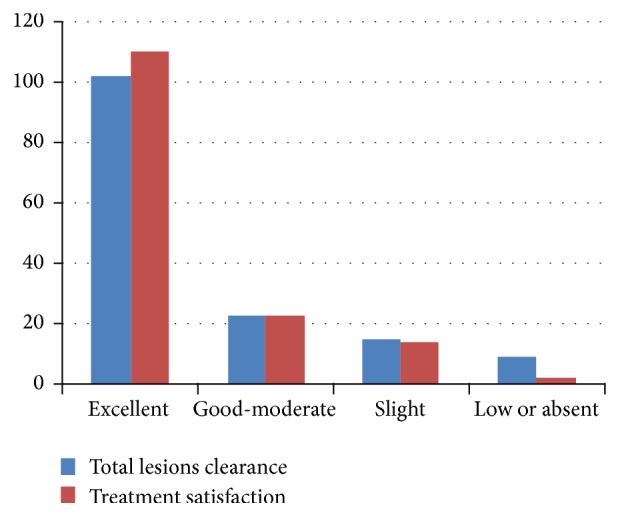
Results.

Relevant side effects, such as blisters, crusts, atrophy, and scars, were absent in all conditions; many patients showed typical FPDL-induced side effects like swelling and purpura, which disappeared three to ten days after treatment. Five patients reported long-lasting purpura (30 days). See Figure 6.

## 4. Discussion and Conclusion

Although intense flash-lamp pulsed-dye laser (FPDL) is normally used in vascular malformation therapy, we treated patients with a number of nonvascular indications.

The study evidences an overall patient improvement that may represent a valid alternative to other procedures.

A correct selection of patients using FPDL is the most useful strategy, as this technique is beneficial in selected patients, such as those with persistent and/or recalcitrant dermatological disorders with vessel involvement. Pediatric population, for example, may become alarmed by treatment and refuse painful procedures resulting in poor patient compliance [14]. Cardiopathic patients, subjects using anticoagulant drugs or unable to receive anesthesia, have great difficulty in undergoing surgical treatment and are thereby more likely to be candidates for FPDL treatment. Lastly, surgical devices are not recommended in certain areas, such as the face, neck, nose, nasal wings, groins, and anogenital areas, due to the risk of disfiguring scars or keloids.

We believe that the success of FPDL treatment lies in the fact that most of the lesions mentioned contain a large number of dilated blood vessels which represent the target of the device. The mechanism of action of FPDL is thereby based on specific destruction of abnormal vessels, components of the lesions themselves (angiokeratoma circumscriptum and striae rubrae), or a selective thrombosis of vessels with the consequent obliteration of the nutrient supply to the lesions (viral wart, angiolymphoid hyperplasia, Kaposi's sarcoma, and basal cell carcinoma).

Also, authors recently demonstrated that the 585 nm FPDL can decrease fibroblast proliferation and collagen type III deposition and furthermore may induce apoptosis and upregulation of extracellular signal-regulated kinase and p38 mitogen-activated protein kinase activity. Its use may be therefore extended to other dermatological conditions such as keloids [15–18].

In this study, similar to previous studies [19], different vascular or vascular dependent lesions were treated using FPDL laser alone or in combination with pulsed CO_2_ laser. FPDL in all cases was reported to be a safe, well-tolerated, and effective treatment method and may be considered an alternative or a complementary treatment for resistant and/or recalcitrant lesions or when contraindications do not suggest the use of other therapies.

Its use, however, should be limited to selected cases in which labelled therapies have not proved effectiveness or when patients are unable to undergo such treatments. Also, off-label procedures must always be carried out carefully, with a strict follow-up in order to ensure safety.

The high cost of the procedure may represent a limit in its use, despite the excellent aesthetic outcome of results.

Future multicentrical studies on single indications, however, are desirable, with possible harmonization of the methods and parameters used.

## Figures and Tables

**Table 1:** Specific PDL approaches for the different cutaneous disorders.

Type of lesions	Patients	Sessions of PDL	Parameters	Combination with CO_2_ laser
Angiokeratoma circumscriptum	5	3	Wavelength 595 nm Energy 6-7 J/cm^2^ Spot size 12 mm Pulse 0.5–1.5 ms	X

Jessner-Kanof disease	4	4	Wavelength 595 nm Energy 6-7 J/cm^2^ Spot size 12 mm Pulse 0.5–1.5 ms	

Viral warts	54	3–7	Wavelength 595 nm Energy 6-7 J/cm^2^ Spot size 12 mm Pulse 0.5–1.5 ms	

Striae	10	4–6	Wavelength 595 nm Energy 6-7 J/cm^2^ Spot size 12 mm Pulse 0.5–1.5 ms	

Angiolymphoid hyperplasia	3	3	Wavelength 595 nm Energy 6-7 J/cm^2^ Spot size 12 mm Pulse 0.5–1.5 ms	X

Superficial and nodular basal cell carcinoma (BCC)	70	5	Wavelength 595 nm Energy 6-7 J/cm^2^ Spot size 12 mm Pulse 0.5–1.5 ms	

Kaposi's sarcoma	3	4	Wavelength 595 nm Energy 6-7 J/cm^2^ Spot size 12 mm Pulse 0.5–1.5 ms	

**Table 2:** Global improvements.

Score 1	Score 2	Score 3	Score 4
Low or no removal of their lesion	Slight clearance	Moderate-good clearance	Excellent clearance
9 (6%)	15 (10%)	23 (15.4)	102 (68.4%)

**Table 3:** Subjective evaluations show that the vast majority of subjects were satisfied or very satisfied.

Unsatisfied	Not very satisfied	Satisfied	Very satisfied
2 (1.3%)	14 (9.3%)	23 (15.4%)	110 (73.8%)
